# Strategic use of male alternative reproductive tactics in cooperatively breeding banded mongoose groups

**DOI:** 10.1098/rsos.242215

**Published:** 2025-02-26

**Authors:** Graham Birch, Hazel J. Nichols, Francis Mwanguhya, Jonathan D. Blount, Michael A. Cant

**Affiliations:** ^1^ Centre for Ecology and Conservation, Faculty of Environment, Science and Economy, University of Exeter, Penryn Campus, Cornwall TR10 9FE, UK; ^2^ Department of Biosciences, Swansea University, Singleton Campus, Swansea, SA2 8PP, UK; ^3^ Banded Mongoose Research Project, Queen Elizabeth National Park, Kasese, Uganda

**Keywords:** life history, cooperative breeder, alternative reproductive tactics, reproductive costs, male, mate choice

## Abstract

Alternative reproductive tactics (ARTs) allow less competitive individuals to reproduce by avoiding direct fights through sneaky strategies. Within cooperatively breeding groups ARTs are rarely reported, potentially owing to observational difficulties or reproductive suppression by group members. In societies where mating opportunities cannot be monopolized by one male, young males could use sneaky tactics as an intermediate ‘stepping-stone’ tactic to gain limited reproductive success while growing in resource-holding potential (RHP). Using decades of pedigree, weight, group demography and behavioural data, we investigated the use of sneaky ‘sneaker’ ARTs in wild male banded mongooses. In this species, groups typically contain more adult males than breeding females, leading to intense male–male competition. Instead of as a stepping-stone, sneaking tactics were typically used by males who had been displaced from mate-guarding status by stronger rivals. Additionally, sneakers had lower siring success compared with mate guards, despite similar weight loss costs, which may explain why males typically avoided reproductive activity entirely rather than sneaking. However, young sneakers gain access to older, higher fecundity females in the group and sneaking may even facilitate inbreeding avoidance. Overall, ARTs in stable social groups can predictively emerge from changes in relative RHP and social status over the lifetime.

## 1. Introduction

In animal societies, male reproductive success is often skewed towards those males with the highest resource-holding potential (RHP) [1,2], defined as a measure of ‘absolute fighting ability’ [3]. These high RHP males are able to monopolize the best territories [4,5], more successfully guard females [6,7] or defend resources that attract females [8,9]. A high RHP is associated with prime-age classes [4,10], a large body size [11] or effective weaponry [12], all of which provide an advantage in competing for mates. Where high RHP individuals can outcompete other males through fights, there may be the selection for low RHP individuals to adopt sneaky alternative reproductive tactics (ARTs) [13]. ARTs are discrete reproductive phenotypes present in the same population [14]. Compared with fighters who directly compete to monopolize access to mates, ARTs often employ parasitic or sneaky tactics. Sneaky tactics can give lower RHP males opportunities for reproductive success through means that avoid the costs of direct confrontation with superior rivals [15–21].

ARTs are taxonomically widespread and take many forms including differences in behaviour or morphology [14,22]. Sneaky morphotypes can be fixed from birth by genes [13,23–25], such as small and large morphs of the pygmy sword tail fish *Xiphophorus nigrensis* [26]*,* from parental effects, such as in spider mites *Tetranychus urticae* [18], or by the developmental environment [27–29] such as the effect of temperature on growth on ART expression in the male squid *Uroteuthis edulis* [19]. Fixed ARTs are typically under negative frequency-dependent dynamics [30–32], meaning that they are more successful when rare in a population. Good evidence in support of negative frequency-dependent dynamics is found in male blue gill sunfish (*Lepomis macrochirus*), where the fitness pay-off of sneaking morphs decreases as they become more common [33].

In contrast to fixed morphologies many ARTs, including sneaky tactics, can be conditionally expressed [30,34,35]. Individuals may strategically choose the best-suited tactic given their relative success based on the current environment and their own condition. For example*,* the success of two distinct sneaky ARTs in ornate tree lizards (*Urosaurus ornatus*) is determined by food availability triggering adaptive tactic switching in males [36]. Flexible use of sneaky tactics is often described as a ‘best-of-a-bad lot’, used by lower RHP males in the population who are at a competitive disadvantage and who would not have success fighting directly with rivals [30,37,38]. By opening up opportunities when at a competitive disadvantage, the strategic adoption of sneaky ARTs could increase lifetime reproductive fitness [39,40].

One strategic use of sneaky tactics is as an ontogenetic ‘stepping stone’. In this pattern of ART adoption, young males with lower RHP make the most of their temporary ‘bad lot’ by initially using sneaky tactics to gain early reproductive success before transitioning to fighting tactics only when they have grown sufficiently to compete successfully with rivals [30,31,41–43]. In the cichlid *Lamprologus callipterus,* small sneakers as they grow eventually become territorial nest builders to attract females themselves [44,45]. Similarly, young African striped mice (*Rhabdomys pumilio*) may use roaming tactics until they grow large enough to defend a harem of females [41,42]. These ‘stepping-stone’ dynamics may be driven by reproductive costs, as lower RHP males are more prone to injuries in fights with superior rivals and may lack the condition required to sustain the energetic demands of the reproductive activity compared with their higher RHP rivals [46,47]. Sneaky tactics may avoid these costs, such as in the two-spotted spider mite (*T. urticae* Koch), where young males increase their survival when using sneaky as opposed to fighting reproductive tactics [48]. The use of sneaky tactics as a stepping stone allows males to gain early reproductive success by avoiding the costs of fighting superior rivals, while positioning themselves for a future transition to more profitable fighting tactics.

Sneaky ARTs may also allow low-RHP males who have been displaced entirely from reproductive positions to make the best of their bad lot by recovering access to reproductive opportunities [37]. For example, in Seba’s short-tailed bats (*Carollia perspicillata*) males who defended their own harems in the past may settle for peripheral roles as they senesce, suggesting these old males use sneaky ARTs as a best-of-a-bad lot option for reproductive success after displacement by rivals [31]. Males may also be displaced from higher to lower quality mating opportunities, such as from high to low fecundity females [49], but these males regain access by using sneaky ARTs. Overall, sneaky ARTs may provide males with a route to increase their reproductive fitness when attempts to fight fail owing to competition.

Here, we describe and evaluate the strategic use and adoption of sneaky ART’s in cooperative breeding banded mongoose (*Mungus mungo*) groups. Male competition is highly costly in cooperative breeding species with fatalities in intragroup fights common [1,50,51]. In many cooperative breeding species, a dominant pair typically suppresses the reproduction of other mature group members [52–54], while maturing males are often forced to disperse to reproduce [2,55], or wait to inherit breeding positions [56]. Instead, in banded mongooses secondary reproducing males that use sneaky ARTs are tolerated in intermediate reproductive skew groups where multiple males and females breed (typically 1−5 females and 3−7 males) [57]. Banded mongooses groups are highly male skewed, typically with multiple mature males for every breeding female [58]. Groups reproduce around four times per year [58]. Dominant ‘guarding’ males can only monopolize reproduction from one female at a time over the duration of short synchronous group oestrus events. Guards follow females continuously while aggressively preventing copulations from rivals [59]. Similar guarding and territorial activity have been associated with costly energetic demands [60,61], and males may suffer opportunity costs from reduced foraging time. Guarding follows assortative mating patterns, with the oldest males guarding the oldest most fecund females, resulting in the top three males siring the majority of the group’s offspring [62]. ‘Sneakers’ attempt to sneak copulations when guards become distracted or during attempts by females to escape their guard [59]. We have historically referred to these sneaker males as ‘pesterers’ [62] because they follow the mate-guard and female around, sometimes interfering with matings. However, to avoid duplicating terms and for consistency with previous work on ARTs we refer to these males here as ‘sneakers’ throughout. Females may attempt to avoid their guard [59] or sneaker males, and there is evidence that females exercise mate choice to avoid inbreeding [63]. Many other mature males are reproductively inactive, displaying neither guarding nor sneaking behaviour [58,62], which we refer to throughout as subordinates.

Our first aim was to test the hypothesis that previously inactive males use sneaky tactics as an intermediate stepping-stone strategy to gain matings while waiting to grow in size, RHP and experience. To examine the social and developmental context in which males switched to sneaky ART’s we compared tactic transitions of inactive and reproductively active males. If sneaking is used as a stepping stone, we predicted reproductively inactive subordinates would more often transition to sneaky tactics then guarding tactics. We then assessed the relative costs and fitness benefits of ARTs in male banded mongooses by comparing weight loss and siring success between mate guarding and sneaking tactics. As a ‘best of a bad lot’ tactic, we expected sneakers to have less access to copulations than guards; hence, we predicted that sneakers would have reduced siring success compared with guards. Finally, on the grounds that ARTs are often expected to exhibit negative frequency-dependent dynamics, we predicted sneaking ARTs will have a higher pay-off when rare.

Our secondary aim was to investigate the hypothesis that the use of sneaky ARTs offers a strategic advantage to males in terms of mate choice. For male mate choice to evolve, males should be forced into time-limited simultaneous assessments over females that vary in aspects of quality such as fecundity [64,65]. To make the best use of their time, high RHP fighters should pursue the most in-demand fecund females, displacing low-RHP rivals to less fecund females. The patterns of mate choice that result where the quality of males and females is matched is known as assortative mating [49]. In banded mongoose groups, males face a time limitation on choice owing to multiple females synchronously breeding during short oestrus events preventing a single male monopolizing all reproductive opportunities, and this probably underlies the assortative mating dynamics seen in the system [62]. Low RHP males, by sneaking, could gain access to higher quality females than they could if they tried to monopolize them directly as a guard. Furthermore, a second assessment for mate choice is genetic compatibility [66]. Same-sex banded mongoose group members are highly related to each other owing to budding dispersal: same-sex bands originating from the same group form new groups [57]. Support for mate choice in banded mongooses by mate guards and reproducing females to avoid inbreeding depression has been found in the past [63,67]. Indeed, males are regularly observed ignoring certain females during oestrus events, while females have been observed attempted to escape from certain guards [59] or sneaker males, which may both be explained by attempts of each sex to avoid inbreeding. If sneaky ARTs open up male mate choice options they may give an advantage accessing less related females and avoiding inbreeding. ARTs may be useful for females who gain access to males they would otherwise be prevented from mating with, as suggested by previous observations of females attempting to escape their mate guards [59]. We assessed whether sneaking tactics provide males with increased opportunities for mate choice in our banded mongoose population. We predicted that mate guards will be more restricted in their access to mates, and guard a more limited pool of females, compared with those that sneaking males pursue. We also tested whether sneaky males have access to less closely related females, suggesting that this tactic may yield benefits to both males and females in terms of inbreeding avoidance.

## 2. Methods

### 2.1. Study population

The banded mongoose study population is located on Mweya Peninsula, Queen Elizabeth National Park, Uganda (0°12′ S, 29°54′ E). The history of each individual and group is known through life-history data collection ongoing since 1995 [57]. For identification, individuals are given a unique fur shave pattern on their back. Data used in this study are from the period between April 2003, where reproductive behaviour data collection started and February 2021.

### 2.2. Data collection and processing

#### 2.2.1. Reproductive tactic data

Groups were visited everyday during the pup care period, which immediately proceeds oestrus. The start of oestrus was identified by the first observations of males pursuing females. Where signs of oestrus were found, groups were visited every day until reproductive behaviour ceased. Each day during oestrus every breeding female received independent observation (focals) lasting 20 min. During focals, any males that guarded the focal female or sneaked were noted. Guards were identified as a single male that followed the focal female within 5 m throughout the 20 min, and sneakers as any male that tried to interrupt the focal-guard pair or make attempts to copulate with the focal female if the guard was distracted. Focals took place during peak foraging periods in the morning and were paused where view of the focal female was obscured or during group alarms. The consistency between focals observations and behaviour outside of these sampling periods has been informed by following these groups for 5 h a day for the last 17 years (100K+ hours of observations in total). Groups continued to be visited every day to collect focals until reproductive behaviour ceased. To avoid sampling immature males, data on males younger than subadults (less than 180 days of age) was removed from all analyses. In each group, there was on average 3.27 guards, 2.77 sneakers and 11.6 inactive males.

Most oestrus events (events spanning the start of oestrus until reproductive behaviours cease) had multiple days of data collection (mean: 2.997, interquartile range (IQR): 3). On each day males were noted as a guard or sneaker according to their behaviour towards females, and as inactive if they showed no interest. For the purposes of examining behaviour transitions between oestrus events, male reproductive behaviour from all data collection days was summarized into one tactic for each oestrus event. To account for any inconsistencies in observations of reproductive behaviour over different data collection days, such as because of missed behaviours or changes in available females, if males were observed to guard on any day of the oestrus event they were defined as a guard, and if they were observed sneaking on any data collection days they were defined as a sneaker. If a male expressed both tactics on separate days they were defined as the more frequent reproductive activity (most days expressed). Males that guarded on an equal number of days to sneaking were defined as a sneaker (if guarded > 50% of days then guard, ties = guard, sneaked > 50% of days then sneaker). In 18.4% of cases where a male was defined as a sneaker, the male guarded on at least 1 day. All other cases sneakers only showed sneaking behaviour. In 14.7% of cases where a male was defined as guard, the male sneaked on at-least 1 day. Males that were inactive for the duration of the oestrus event were recorded as a subordinate. We assumed that these males defined as subordinates did not engage in unobserved reproductive activity, which would be a possibility if this activity occurred in dense vegetation or outside of visiting periods of observers to the group.

#### 2.2.2. Age, weight and demographic data

All males in this analysis were followed from birth to death and are therefore of a known age. We calculated the sex-specific age ranks of all individuals in each group as a measure of relative RHP to competitors. Many males were of the same age having been born in the same litter. In our analysis shared age ranks were assigned to the minimum rank and subsequent younger males ranks left a gap, for example, if there were three age rank two males the next oldest was assigned age rank 5.

Body weights have been collected for the population since 2000. Weight collection frequency varies between individuals and groups depending on their degree of habituation. To account for this variation, weights were averaged over a shared time period. For the tactic transition and paternity analysis, weights were averaged for the 60 days before and after a given oestrus event (oestrus weight). The mean number of weights included in this average was 7.9 (lower quartile = 2.6, upper quartile = 11.1). To control for variation in male weights between groups we centred weight around the group mean (individual oestrus weight – mean male oestrus weight). A 6.6% (239 out of 3638) of oestrus weights could not be calculated with the data available. Imputation has been carried out in previous studies on long-term populations where missing data can occur [68,69], including this same population of banded mongooses [70]. Using the full history of weight collection for each individual, and the correlation between age and weight in banded mongooses, these missing weights were inputted (see the electronic supplementary material for more detail).

Predicting the likely weight of males through imputation methods is not applicable to the measurement of actual weight loss; therefore, for weight loss models prior and post-oestrus, weights were extracted separately. Prior oestrus weights were averaged over the 60 days prior to each oestrus event (mean number of weights = 4.7, lower quartile = 3, upper quartile = 5.9) and the post-oestrus weight was the nearest single weight recorded after an oestrus event ceased. A preliminary model was run to detect an effect of time to post-oestrus weight collection on weight loss, of which no significant effect was found when restricting sampling to 9 days post-oestrus (mean = 2.43 days, lower quantile = 0 days, upper quantile = 4 days). Males with missing weights were simply not used.

#### 2.2.3. Sired litters and group-centred relatedness

A genetic pedigree has been collected since 2003 (see references for how the pedigree is obtained [11,40]). The methods for constructing the genetic pedigree and calculating pairwise relatedness for the banded mongoose population have been described previously [71]. A banded mongoose’s gestation period is around nine weeks (pooled data from [58,65]). Fifty-seven litters could be connected to 35 mothers that received attention from at least one sneaking and guarding male in oestrus events approximately nine weeks prior to the litters birth date (ended 59 ± 15 days before). This included 60 cases of successful sires (three had multiple paternity) from interactions with 104 individual males

Group-centred relatedness was calculated by taking the average pedigree relatedness of a given male to every adult female present in the same group during an oestrus event, and then for each male, the above average was deducted from pedigree relatedness to each adult female present in the group.

### 2.3. Statistical analysis

All models were fitted using Bayesian inference (JAGS MCMC) in R [72,73]. To improve model convergence, numeric covariates with a range below 0 and above 1 were standardized. Uninformed priors were fitted to all models. Multicollinearity was checked using cross-correlation plots from the ggmcmc package [74]. Chain convergence was checked using the values from the JAGS model output, with all models showing convergence of chains for each fitted parameter (*R* < 1.1). Convergence was also checked using traceplots by eye (ggmcmc). Credibility was defined for where 97.5% or more of an effect posterior was on one side of zero.

#### 2.3.1. Analysing the adoption of alternative reproductive tactics using tactic-transition models

The transition probability of males from one reproductive tactic to the next was modelled using JAGS MCMC [31,72]. This model was based on 3638 tactic transitions of which 639 involved transitions to or from a sneaking tactic. The model used state matrix **z** with element **z***_i,t_* for the tactic of male *i* at oestrus event *t,* where *i* comprises the 320 males that have been followed from birth to death in the study population, *t* comprises each oestrus event in order a given male has participated in throughout their life. A state-transition matrix **Ω** was set with four dimensions, previous reproductive tactic *n*, new state *m*, male *i* and oestrus event *t*. The state process **ω***_n,m,i,t_* represents the probability that the male at reproductive tactic *n* at oestrus event *t*, will be in state *m* at the next oestrus event *t +* 1. The probability of leaving any reproductive status and dying before the next oestrus event was defined as 1 minus the reproductive status-related survival probability. Once a male had died its probability of remaining dead was defined as 1. Since we have complete records of group composition during oestrus events; therefore, each male *i* had reproductive tactic data for each oestrus event *t* they lived through, an observation matrix was not required (always observed). Using the tactic-transition matrix **Ω** reproductive tactic transitions (iterations = 20 000, thinning interval = 100, burn in = 2500 and chains = 3) were regressed against age and weight relative to other males in the group (age rank and group-centred weight) as a metric of RHP (metrics of RHP). Running separate models for moderately correlated variables may lead to bias exaggerating their significance, justifying age rank and group-centred weights (*r* = 0.44) inclusion in the same model [75]. Interactions between group-centred weight and age rank were however not fitted as issues with multicollinearity would be exaggerated [75]. Group sex ratio was fitted in the same model (number of males over the number of females of at least 1 year of age) to test for an effect of competition. Interactions between group sex ratio and both age rank and group-centred weight were fitted but were removed when interaction terms proved not credible. To control for common group membership during oestrus events and repeated sampling on the same males, random effects for oestrus event ID (*n* = 375), group ID (*n* = 20) and male ID (*n* = 320) were fitted.

#### 2.3.2. Modelling the weight loss costs of alternative reproductive tactics

Five-hundred and fifty-four weight changes from subordinates, 170 weight changes from sneakers and 265 weight changes from guards were included. To control for common group membership during oestrus events and repeated sampling on the same males, random effects for oestrus event ID (*n* = 118), group ID (*n* = 12) and male ID (*n* = 259) were fitted. Percentage weight loss for each individual over an oestrus event was normally distributed, and as such was regressed with the behavioural tactic (subordinate versus guard) using a Gaussian distribution in JAGS MCMC (iterations = 50 000, thinning interval = 100, burn in = 5000 and chains = 3).

#### 2.3.3. Modelling the reproductive success of alternative reproductive tactics

Thirty-eight percent of all cases where a female successfully produced a litter saw the female receive attention from at least one sneaker male along with a mate guard, as opposed to just receiving attention from a mate guard. Fifty-seven of these litters could be attributed with high confidence to a sire observed to give attention to the female as a sneaker or guard verified through pedigree data taken from 2003 to 2019, see [62,76] for methodology. These 57 l were produced by 35 individual females who interacted with 104 individual males during 41 unique oestrus events, all included as random effects. For each dyad per oestrus event, a binomial success or fail was determined depending on whether the male succeeded in siring offspring. The probability that a given male would successfully sire was modelled using a Bernoulli generalized linear mixed model (GLMM) fitted in JAGS MCMC (iterations = 100 000, thinning interval = 100, burn in = 10 000 and chains = 3). The model was fitted with the reproductive tactic of each male to test whether guards were more successful than sneakers (guards as 1, sneakers as 0), and the proportion of competitors adopting the same tactic who interacted with the same female. To control for the fact that each new male interacting with a female decreased the chance each male had of siring her offspring, the number of male competitors interacting with each female over the course of the oestrus event was fitted as a fixed effect (mean = 2.66, lower quantile = 1, upper quantile = 3). To assess if the effect of reproductive tactic was influenced by the proportion of competitors that shared the same tactic, interaction terms were initially included, but dropped from the final model when they were not proved credible.

#### 2.3.4. Modelling evidence alternative reproductive tactics use is advantageous for male mate-choice

For each day where a male over 180 days of age has interacted (as a guard or sneaker), a binomial response variable was added for each female present in the group to distinguish the specific females the male interacted with. This included 1558 guarding and 674 sneaking interactions involving 228 unique adult males and 230 unique adult females in 297 oestrus events. The smaller number of oestrus events and males compared with the tactic transition analysis is because of only including interactions where the relatedness of the male and female were known, as well as the age of the female involved. Only rarely did a given male interact with multiple females on the same day (308 out of 2232, approx. 14% of cases), yet as different groups of females enter and leave oestrus across multiple days males commonly (927 out of 1298, approx. 71.4% of cases) interacted with multiple potential mates over the course of the oestrus event. The probability that a given male would interact with a female was modelled using a Bernoulli GLMM fitted in JAGS MCMC (iterations = 20 000, thinning interval = 100, burn in = 2000, chains = 3). This was regressed with group-centred relatedness, and an interaction between male and female age rank to assess patterns of assortative mating in banded mongooses. Furthermore, interactions with reproductive tactics were fitted, a three-way interaction with male and female age rank and two-way with relatedness, respectively, to assess whether guards and sneakers have different preferences for females. The interaction between relatedness and tactic was dropped when it did not prove credible. To account for the probability of interacting being inversely associated with the number of females, the number of adult females present in the group was added as a fixed covariate.

### 2.4. Ethics

Prior approval of all work was received from the Uganda Wildlife Authority (UWA) and the Uganda National Council for Science and Technology (UNCST). The ethical review committee of the University of Exeter approved all research activities.

## 3. Results

**Figure 1.**
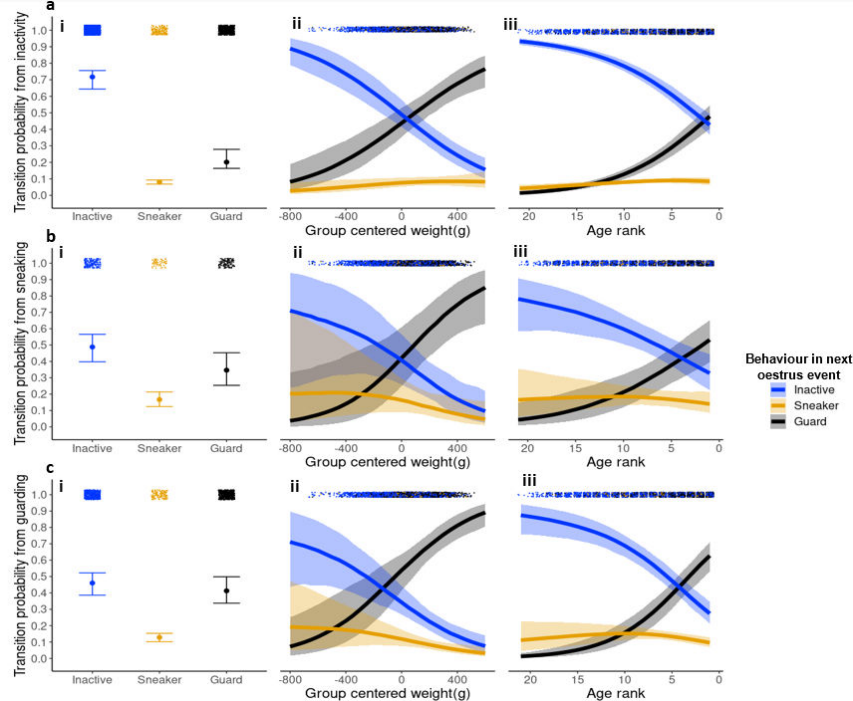
The effect of group-centered weight (ii) and age rank (iii) on between-oestrus event state transition probabilities. Lines represent the mean posterior probability and ribbons are 95% credible intervals. Each panel represents transition probabilities from the three distinct states: from inactivity (a), from sneaking (b), and from guarding (c). Black lines and ribbons correspond to transitions to guarding, orange transitions to sneaking and blue transitions to subordinate tactics. Points at the top of each panel show raw recorded transitions to each tactic. Mean (points) and credible intervals (error bars) for all nine transitions for reference based of a null model with no covariates are shown in (i).

Transitions to sneaking tactics were rare overall (figure 1a(i)*,* b(i)*,* and c(i)). On average, inactive subordinates transitioned into guarding tactics (mean = 0.2, high credible interval (hci) = 0.28, low credible interval (lci) = 0.16) more often than into sneaking tactics (mean = 0.08, hci = 0.09, lci = 0.07). Sneaking was the rarest transition from a guarding tactic (mean = 0.13, hci = 0.15, lci = 0.1), with keeping a guarding tactic (mean = 0.41, hci = 0.5, lci = 0.34) statistically similar to dropping into a subordinate tactic (mean = 0.46, hci = 0.52, lci = 0.39). Sneaking tactics themselves were unstable having a lower probability of being retained (mean = 0.17, hci = 0.21, lci = 0.12) than gaining a guarding tactic (mean = 0.35, hci = 0.45, lci = 0.25), with dropping into a subordinate tactic being the most likely transition (mean = 0.49, hci = 0.57, lci = 0.4). Transition probabilities from a sneaking tactic resemble transition probabilities from a guarding tactic (figure 1b,c), suggesting sneakers and guards are functionally similar in their future use of tactics.

Age rank and group-centred weight were correlated (*r* = −0.44), and the effect of increasing weight on transition probabilities largely mirrored the effect of decreasing age rank (figure 1(ii,iii)). In all reproductive tactics, there was a negative effect of age rank (older) on transitions to subordinate, and positive (younger) to a guarding tactic (electronic supplementary material, table S1a). Therefore, as males aged, they became more likely to adopt a future guarding tactic and less likely to adopt a subordinate tactic (figure 1(iii)). Similarly, there was a credible negative effect of weight on transitions to subordinate, and a positive effect of weight on transitions into guarding tactics (figure 1(ii)). As males moved into older age rank and grew in weight, their probability to transition into a sneaking tactic from all reproductive tactics credibly decreased (electronic supplementary material, table S1a), with the exception of weight having no credible effect on the subordinate to sneaking transition probabilities. Posterior probabilities to transition to a sneaking tactic (figure 1) only started to decline from a peak at intermediate age ranks (from subordinate: age rank 5, mean = 0.09, hci = 0.11, lci = 0.08; from sneaker: age rank 11, mean = 0.19, hci = 0.26, lci = 0.23; from guard: age rank 10, mean = 0.15, hci = 0.2, lci = 0.11) and weights (from sneaker: −250 g, mean = 0.19, hci = 0.3, lci = 0.12; from guard: −200 g, mean = 0.16, hci = 0.23, lci = 0.1), continuing to decline towards age rank 1 (from subordinate: mean = 0.09, hci = 0.11, lci = 0.06; from sneaker: mean = 0.14, hci = 0.21, lci = 0.09; from guard: mean = 0.1, hci = 0.13, lci = 0.07) and the heaviest weights e.g. +400 g (from sneaker: mean = 0.09, hci = 0.18, lci = 0.04; from guard: mean = 0.06, hci = 0.1, lci = 0.03).

The most likely transition for young males was remaining or becoming an inactive subordinate, but if young inactive males (below age rank 19) did become active they transitioned into sneaking tactics more often than to guarding tactics (to sneaker: mean = 0.05, hci = 0.07, lci = 0.03; to guard: age rank 20, mean = 0.02, hci = 0.03, lci = 0.01). In comparison, slightly older guards, younger than age rank 17, were more like to transition into a sneaking tactic than to keep their guarding tactic (age rank 18 to sneaker: mean = 0.13, hci = 0.22, lci = 0.06; to guard: age rank 20, mean = 0.03, hci = 0.06, lci = 0.01). As individuals move into older age ranks, transitions to a guarding and sneaking tactic became statistically similar until guarding transitions overtook sneaking transition probabilities at age rank 9 in inactive subordinates (to sneaker: mean = 0.08, hci = 0.09, lci = 0.07; to guard: mean = 0.13, hci = 0.16, lci = 0.1), and at age rank 7 in guards (to sneaker: mean = 0.15, hci = 0.18, lci = 0.12; to guard: mean = 0.28, hci = 0.36, lci = 0.2).

Sex ratios were on average male biased (mean = 2.2 males per female IQR = 1.4). There was no credible effect of biased sex ratios on any tactic transition probabilities (see the electronic supplementary material, table S1a and figure S3 for more detail).

Sneakers lost a similar amount of weight (electronic supplementary material, figure S1; mean = −2.3% bodyweight, hci = −3.37%, lci = −1.28%) to guards (mean = −2.31%, hci = −1.47%, lci = −3.21%) over the course of an oestrus event, over subordinates that had a statistically neutral weight change (mean = 0.32%, hci = 1.11, lci = −0.48).

Although on each day a given male is usually recorded interacting with a single female, most males interacted with multiple females over the course of the oestrus event, with sneakers on average interacting with more females (mean females interacted with per day of oestrus: guards = 1.11 ± 0.01 s.e.m., sneakers = 1.18 ± 0.02 2 s.e.m.; and mean females interacted with over each oestrus event: guards = 2.34 ± 0.052 s.e.m., sneakers = 2.71 ± 0.05 s.e.m.).

Guards had a credibly higher (mean = 0.4, hci = 0.52, lci = 0.28) chance than sneaking competitors (mean = 0.12, hci = 0.19, lci = 0.6) to sire a female’s offspring (electronic supplementary material, table S1a). There was no credible interaction between the number of competitors and tactic (guard versus sneaker) on success at siring offspring, with guards maintaining a siring advantage against single sneakers (electronic supplementary material, figure S2—guard: mean = 0.65, hci = 0.80, lci = 0.46 versus sneaker: mean = 0.27, hci = 0.42, lci = 0.14) or multiple sneakers, e.g. 2 (guard: mean = 0.67, hci = 0.83, lci = 0.47 versus sneakers: mean = 0.13, hci = 0.54, lci = 0.23).

There was a credible negative effect of the proportions of competitors using the same tactic to compete for a female on the chance to sire her offspring. However, there was no evidence of an interaction with tactic, suggesting the success of sneaking and guarding males are under similar negative frequency-dependent dynamics. For example, when controlling for competition and given a scenario where three males interact with the same female, independent of the tactic adopted, the two males adopting the same tactic have a relative disadvantage (mean = 0.26, hci = 0.4, lci = 0.15) to the lone male adopting the rarer tactic (mean = 0.48, hci = 0.66, lci = 0.3).

**Figure 2.**
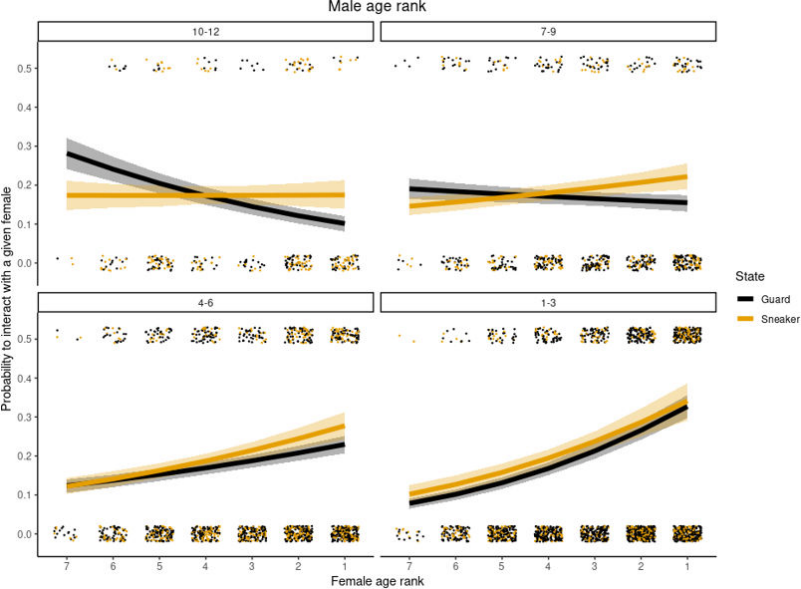
Probability that males interact with females of different age rank, for four different age categories of male, from oldest ranked males (top left) to youngest (bottom right). Black lines are guards, orange lines are sneakers. Lines represent the mean posterior probability and ribbons 95% credible intervals. Dots show raw cases of interactions (*y* = 0.5) and absence of an interaction (*y* = 0) between a male and a female of given age ranks. Posterior probabilities have been averaged for age rank bands of three for plotting purposes. To control for amount of choice, these probabilities are modelled given there are seven females present in the group.

Guarders assortatively mated according to their age rank, compared with sneaker males that continued to interact with older females at younger age ranks. There was a credible interaction between tactic (guard or sneaker), and male and female age rank on the probability a male interacted with a given female (electronic supplementary material, table S1d). Males at the oldest age ranks (1–3) typically interacted with the oldest females more often regardless of their reproductive tactic. For example, given seven females in the group, guards and sneakers interact with age rank 1 females around a third of the time (figure 2: tactic = guard, mean = 0.33, hci = 0.36, lci = 0.3 versus tactic = sneaker, mean = 0.34, hci = 0.39, lci = 0.29), compared with age rank 7 females around 1 in 10 times (tactic = guard, mean = 0.08, hci = 0.09, lci = 0.06 versus tactic = sneaker, mean = 0.1, hci = 0.12, lci = 0.08). Guarding and sneaking interactions converge at older age ranks. Guards continued to assortatively mate at younger age ranks, with the youngest males interacting with the youngest females more often. For example, if there are seven females in the group, males of age ranks 10−12 were far more likely to interact with age rank 7 females (mean = 0.28, hci = 0.32, lci = 0.24) than the top females (i.e. age rank 1:, mean = 0.1, hci = 0.12, lci = 0.08). Although younger sneakers did interact with the top females less than older sneakers, this switch in interaction probability towards younger females noticeably lagged behind guards (figure 2), with the youngest sneakers such as age ranks 10−12 continuing to interact with the top females as often as the youngest females (age rank 1 females:, mean = 0.17, hci = 0.21, lci = 0.14 versus age rank 7 females: mean = 0.17, hci = 0.21, lci = 0.14). This suggests sneakers interacted with older females more than would be expected if they adopted a guarding tactic.

Males had a preference for less closely related females compared with females with a high relatedness to themselves (electronic supplementary material, table S1; e.g. group-centred relatedness = −0.25, mean given seven females present = 0.21, hci = 0.24, lci = 0.19; group-centred relatedness = 0.35: mean given seven females present = 0.15, hci = 0.17, lci = 0.13). There was no interaction between reproductive tactics and relatedness, suggesting avoidance of inbreeding does not differ amongst guards and sneakers. There was also no interaction between a male’s age rankand a female’s relatedness to interacting males, suggesting younger males were not restricted in their choice for unrelated females as they were for older fecund females.

## 4. Discussion

Our study examined the adoption, the costs and benefits associated with two ARTs in banded mongooses, mate guarding and sneaking. The adoption of sneaking tactics by previously inactive males was consistently rarer than guarding, suggesting that sneaking is not used as a stepping stone as males more often remain reproductively inactive until they can gain mate-guarding roles. Sneaking tactics were also only rarely adopted by guards, but sneaking did increase for low weight and younger previous guards indicating sneaking tactics are used as a rare best of a bad lot option when low RHP males are displaced. Contrary to our expectation that sneaking behaviour would represent a less costly reproductive tactic than mate guarding, we found that sneaking males lost a similar percentage of body weight over the oestrus period as guarding males. Males who did not participate in sneaking or mate guarding had no net weight loss. The weight loss associated with both sneaking and mate guarding is probably driven by a combination of shared physical activity and the opportunity costs of reduced foraging [61,77]. We also found that sneakers had a clear siring disadvantage compared with mate guards, and the probability of successful siring decreased with the proportion of rivals using the same tactic. These siring success results indicate that the fitness benefits of both sneaking and mate guarding exhibited negative frequency-dependent dynamics, consistent with ARTs in other species [13,24,30,33].

The lack of evidence that sneaking tactics are used as a ‘stepping stone’ to future guarding tactics is in contrast to their conditional use by growing males in other systems [30,43–45] before graduating to fighting tactics, e.g. Arctic char (*Salvelinus alpinus*) and pink salmon (*Oncorhynchus gorbuscha*) [78]. Instead, younger, low-RHP male banded mongooses typically stay inactive until they can grow to gain a guarding position rather than adopt sneaking tactics in the interim. One likely reason for the lack of sneaking tactics being adopted by low-RHP males is displacement by other sneakers. Displacement is suggested based on observations of sneakers chasing off other sneakers who approached the same guarded female, explaining the negative frequency-dependent siring success found in this study. A small size may typically be beneficial for sneakers in other systems if it helps males remain undetected by fighters [44], but competition amongst sneakers for access to females would mean that any advantage of a small size may not be realized if males are displaced by higher RHP sneakers. Another reason for the lack of adoption of sneaking tactics is the relatively high cost of sneaking. Whereas using sneaky tactics can reduce reproductive costs compared with fighters in other systems [30], such as mortality costs in the two-spotted spider mite (*T. urticae* Koch) [48], sneaking banded mongooses suffered similar weight loss costs compared with guarding males. These weight loss costs will be felt hardest by younger, low-RHP males who may lack the condition required to sustain the energetic demands of reproductive activity compared with older, higher RHP males in the group [46,47]. Overall, low-RHP male banded mongooses may accrue fewer benefits and incur higher costs of sneaking than is the case for adopters of sneaky tactics in other systems, explaining their lack of adoption as an initial early opportunity for reproductive success on the way to guarding tactics in later life.

The weight loss costs associated with sneaking could also lead to future loss of reproductive success. For example, in African striped mice (*R. pumilio*) males that adopt roaming tactics suffer a delay in the weight gain needed to defend future harems, which can reduce lifetime reproductive success [41,42]. Similarly, in banded mongooses, our transition matrices indicate that subordinates needed to reach a certain weight relative to other group members to transition into mate-guarding tactics. Thus, weight loss by sneakers could potentially hinder their growth and the acquisition of future profitable guarding roles, diminishing their lifetime reproductive fitness. Given the unprofitability of sneaking and mate guarding for young and low-weight males, these individuals may gain from investing in helping instead [79,80].

A rare few young males did transition into sneaky tactics. Why these males did so, despite the above costs, can be understood by considering individual differences. For example, for higher RHP males sneaking is unlikely to have an as determinantal impact on their future reproductive success as they have more resources to maintain reproductive activity and remain competitive, making sneaking a less costly option. There is significant variation in weight in the same age class such as amongst pups, explained by the amount of care they receive from the group as they grow [81]. These heavier pups as they mature should also have more resources to use sneaky tactics without impairing their future reproductive success relative to smaller pups, which may partly explain why heavier subordinates more often transition into sneaky tactics than smaller subordinates. The use of sneaky tactics early on may contribute to higher life-time reproductive success where individuals have the relative condition for sneaky tactics to pay off, such as attained by heavier pups [81].

The use of ARTs should be more common under higher competition. For example, under high population densities African striped mice (*R. pumilio*) increasingly use philopatric inheritance strategies, as males using dispersing strategies have reduced success at gaining their own harems [56]. However, for banded mongooses there was a lack of evidence that groups with higher male sex skew, therefore higher competition, contribute to the adoption of sneaking tactics by either previous inactive or guarding banded mongooses. Yet, since male-biased sex ratios are the norm for banded mongoose groups any potential effect of the number of potential rivals for each female may be overshadowed by RHP differences between individual competitors. Among previous guards, lower weight and younger males in the group had the highest probability to transition into sneaking tactics, suggesting that sneakers were often previous low-RHP guards that had been displaced under these invariably high levels of mate competition.

While sneaking tactics involved substantial costs and were the rarest reproductive tactic (figure 1), we found that by adopting sneaking tactics males removed restrictions on mate choice. Younger guards had lower RHP and were restricted to guarding younger females owing to assortative mating dynamics while the oldest males guarded the oldest females [49]. Assortative guarding interactions align with previous results showing that the oldest male banded mongooses sire the majority of groups’ offspring through copulating with the oldest most fecund females [62]. Sneakers instead interacted with females of age ranks older than would be expected based on assortative mating of mate guards, suggesting that freedom to choose mates in a sneaking tactic improves access to fecund females. A chance to sire from more fecund older females may partially compensate for a reduced chance of siring success compared with more assured siring success guarding lower quality females, while also facilitating access to females in cases where all partners are guarded by higher RHP rivals.

In cooperatively breeding fairy-wrens *Malurus cyaneus* auxiliary males, close kin of breeding individuals that help to raise their offspring, pursue extra-pair copulations when new unrelated females join the group [82], important for cooperative breeders owing to the overall high relatedness between members [83]. Sneaking tactics may similarly facilitate inbreeding avoidance by male banded mongooses who do not hold the main breeding roles. For example, we found both guards and sneaker male banded mongooses displayed a preference for interacting with less closely related females in the group, aligning with previous findings indicating inbreeding avoidance among mate-guarding males [63]. This preference implies that low-RHP guards do not face the same level of constraint in mate choice based on relatedness compared with mate choice based on a females age. Our transition results indicate that lower RHP guards, who may only have had access to closely related females, switch to sneaking tactics and this may allow inbreeding avoidance as a sneaker male instead of settling for reproductive inactivity. Flexible switching to sneaking tactics may explain how guards and sneakers maintain a similar preference for less related females in the group, suggesting indirect evidence of the adoption of sneaky ARTs as an option for inbreeding avoidance.

Females may benefit from the use of sneaking tactics by males. Female mate choice can prevent less attractive males from exercising mate choice, compared with attractive males who maintain multiple partner options who can continue to exhibit choosiness [65,84,85]. In banded mongoose groups, males guard access to females but females can have some control over their partners by making attempts to escape their guard [59]. Although both sexes prefer to avoid close relatives as mates, male-biased sex ratios in groups mean females may often disagree with their guard over partner choice as females have a larger pool of partners to choose from. Additionally, as females are limited in the offspring they can produce, they should be choosier than males who can sire offspring from multiple litters [64,86]. Females may attempt to escape their guard to avoid inbreeding, evidenced by previous work showing that in cases of non-guard sires the successful sire is typically less related to the female than the failed mate guard [63]. Sneakers may act as a convenient alternative partner for females to avoid inbreeding in cases where females disagree with a guard’s advances. Even if the guards have copulated, any successful copulation with sneakers may allow potential cryptic female choice mechanisms to select the most compatible sperm [86,87], which may explain some of the skew towards less related sires found previously [63]. However, whether female choice mechanisms are present in banded mongooses is currently unknown. Sneaking tactics may benefit both males and females by expanding partner options that were previously restricted by guarding males and facilitating inbreeding avoidance.

A characteristic of banded mongoose sneaky ART’s rarely described in other cooperative groups is they are used in reproductive competition with group members rather than sneaking copulations from outside their own group such as in meerkats *Suricata suricatta* [88], prairie voles *Microtus ochrogaster* [89], African striped mice *R. pumilio* [41], or placid greenbuls *Phyllastrephus placidus* [90]. Sneaking within specifically mammalian cooperative groups may be comparatively rare owing to dominant males pre-emptively suppressing subordinate reproduction through threats and violence [52–54,91–94], forcing males to sneak copulations from [88,89] or disperse permanently to [95], another group rather than sneak copulations in their natal groups. Such attempts at reproductive suppression are more feasible where there is only a single breeding female to guard, and hence a single source of mating success for rival males. However, in societies that feature multiple reproductive females, the ability of males to monopolize reproduction is inherently limited. Moreover, the fitness costs and benefits of suppression behaviour, and attempts to evade suppression, are likely to vary systematically over the lifetime depending on kinship dynamics [96,97] as relatedness to females and males in the group changes. In many social animals, we would expect ARTs to be adopted in a flexible manner over the life course depending on individual condition during development and relative competitive ability as shown here. For banded mongooses such flexibility includes delaying reproduction when young and, later in life, adopting sneaker tactics after losing dominant status.

## 5. Conclusion

We did not find evidence that sneaky ARTs are used as a stepping-stone reproductive tactic in male banded mongooses. The costs and depreciating siring success of sneaking may typically select for young, light subordinates to remain reproductively inactive until they have an RHP high enough to successfully adopt a mate-guarding tactic. Instead, sneaky ARTs in male banded mongooses are rare and we suggest are used by a minority of males as a best-of-a-bad lot option if mate-guarding positions are lost or fail to be secured. However, where sneaking tactics are adopted, sneakers take advantage of reduced restrictions on mate choice by strategically interacting with the oldest, most fecund females in the group, suggesting that sneaky ARTs can be used by low RHP males to improve male mate choice options in social groups. We further suggest these sneaky ARTs may facilitate inbreeding avoidance for both males and females. Finally, the presence of sneaky ART’s used by individuals within their own social groups suggests a marked difference between banded mongooses and other mammalian cooperative breeders where dominants pre-emptively supresses the competition.

## Data Availability

Data and relevant code for this research work are stored in GitHub: [98] and have been archived within the Zenodo repository: [99]. Supplementary material is available online [100].

## References

[1] Cant MA, Johnstone RA. 2000 Power struggles, dominance testing, and reproductive skew. Am. Nat. **155**, 406–417. (10.1086/303328) 10718735

[2] Nonacs P, Hager R. 2011 The past, present and future of reproductive skew theory and experiments. Biol. Rev. **86**, 271–298. (10.1111/j.1469-185x.2010.00144.x) 20545672

[3] Parker GA. 1974 Assessment strategy and the evolution of fighting behaviour. J. Theor. Biol. **47**, 223–243. (10.1016/0022-5193(74)90111-8) 4477626

[4] Hyman J, Hughes M, Nowicki S, Searcy WA. 2004 Individual variation in the strength of territory defense in male song sparrows: correlates of age, territory tenure, and neighbor aggressiveness. Behaviour **141**, 15–27. (10.1163/156853904772746574)

[5] Carvalho MRM, Peixoto PEC, Benson WW. 2016 Territorial clashes in the Neotropical butterfly Actinote pellenea (Acraeinae): do disputes differ when contests get physical? Behav. Ecol. Sociobiol. **70**, 199–207. (10.1007/s00265-015-2042-6)

[6] Yasuda CI, Koga T. 2016 Importance of weapon size in all stages of male–male contests in the hermit crab Pagurus minutus. Behav. Ecol. Sociobiol. **70**, 2175–2183. (10.1007/s00265-016-2221-0)

[7] Galeotti P. 1998 Correlates of hoot rate and structure in male tawny owls Strix aluco: implications for male rivalry and female mate choice. J. Avian Biol. **29**, 25. (10.2307/3677337)

[8] Greenfield MD. 2010 Sexual selection in resource defense polygyny: lessons from territorial grasshoppers. In The evolution of mating systems in insects and arachnids (eds JC Choe, BJ Crespipp), pp. 75–88. Cambridge, UK: Cambridge University Press. (10.1017/CBO9780511721946.005)

[9] Kelly CD. 2008 The interrelationships between resource-holding potential, resource-value and reproductive success in territorial males: How much variation can we explain? Behav. Ecol. Sociobiol. **62**, 855–871. (10.1007/s00265-007-0518-8)

[10] Kutsukake N, Clutton-Brock TH. 2008 Do meerkats engage in conflict management following aggression? Reconciliation, submission and avoidance. Anim. Behav. **75**, 1441–1453. (10.1016/j.anbehav.2007.09.018)

[11] Wright E, Galbany J, McFarlin SC, Ndayishimiye E, Stoinski TS, Robbins MM. 2019 Male body size, dominance rank and strategic use of aggression in a group-living mammal. Anim. Behav. **151**, 87–102. (10.1016/j.anbehav.2019.03.011)

[12] Kruuk LEB, Slate J, Pemberton JM, Brotherstone S, Guinness F, Clutton-Brock T. 2002 Antler size in red deer: heritability and selection but no evolution. Evolution **56**, 1683–1695. (10.1554/0014-3820(2002)056[1683:asirdh]2.0.co;2) 12353761

[13] Neff BD, Svensson EI. 2013 Polyandry and alternative mating tactics. Phil. Trans. R. Soc. B **368**, 20120045. (10.1098/rstb.2012.0045) 23339236 PMC3576579

[14] Oliveira RF, Taborsky M, Brockmann HJ. 2008 Alternative reproductive tactics: an integrative approach. Cambridge, UK: Cambridge University Press. (10.1017/CBO9780511542602)

[15] Parker P, Maniscalco JM. 2014 A long-term study reveals multiple reproductive behavior strategies among territorial adult male Steller sea lions (Eumetopias jubatus). Can. J. Zool. **92**, 405–415. (10.1139/cjz-2013-0099)

[16] Dougherty LR, Skirrow MJA, Jennions MD, Simmons LW. 2022 Male alternative reproductive tactics and sperm competition: a meta‐analysis. Biol. Rev. **97**, 1365–1388. (10.1111/brv.12846) 35229450 PMC9541908

[17] Fasel NJ, Wesseling C, Fernandez AA, Vallat A, Glauser G, Helfenstein F, Richner H. 2017 Alternative reproductive tactics, sperm mobility and oxidative stress in Carollia perspicillata (Seba’s short-tailed bat). Behav. Ecol. Sociobiol. **71**, 11. (10.1007/s00265-016-2251-7)

[18] Schausberger P, Sato Y. 2019 Parental effects of male alternative reproductive tactics (ARTs) on ARTs of haploid sons. Funct. Ecol. **33**, 1684–1694. (10.1111/1365-2435.13385)

[19] Pang Y, Chen CS, Kawamura T, Iwata Y. 2022 Environmental influence on life-history traits in male squid Uroteuthis edulis with alternative reproductive tactics. Mar. Biol. **169**, 33. (10.1007/s00227-022-04017-y)

[20] Kustra MC, Alonzo SH. 2020 Sperm and alternative reproductive tactics: a review of existing theory and empirical data. Phil. Trans. R. Soc. B **375**, 20200075. (10.1098/rstb.2020.0075) 33070732 PMC7661440

[21] McCallum ES, Bose APH, Lobban N, Marentette JR, Pettitt-Wade H, Koops MA, Fisk AT, Balshine S. 2019 Alternative reproductive tactics, an overlooked source of life history variation in the invasive round goby. Can. J. Fish. Aquat. Sci. **76**, 1562–1570. (10.1139/cjfas-2018-0340)

[22] Gross MR. 1996 Alternative reproductive strategies and tactics: diversity within sexes. Trends Ecol. Evol. **11**, 92–98. (10.1016/0169-5347(96)81050-0) 21237769

[23] Shuster SM. 2008 The expression of crustacean mating strategies. In Alternative reproductive tactics: an integrative approach (eds RF Oliveira, M Taborsky, HJ Brockman), pp. 224–250. Cambridge, UK: Cambridge University Press. (10.1017/CBO9780511542602.010)

[24] Tomkins JL, Brown GS. 2004 Population density drives the local evolution of a threshold dimorphism. Nature **431**, 1099–1103. (10.1038/nature02918) 15510148

[25] Skwierzyńska AM, Plesnar-Bielak A, Kolasa M, Radwan J. 2018 Evolution of mate guarding under the risk of intrasexual aggression in a mite with alternative mating tactics. Anim. Behav. **137**, 75–82. (10.1016/j.anbehav.2018.01.002)

[26] Zimmerer EJ, Kallman KD. 1989 Genetic basis for alternative reproductive tactics in the pygmy swordtail, Xiphophorus nigrensis. Evolution **43**, 1298. (10.2307/2409364) 28564513

[27] Stockley P, Searle JB, Macdonald DW, Jones CS. 1994 Alternative reproductive tactics in male common shrews: relationships between mate-searching behaviour, sperm production, and reproductive success as revealed by DNA fingerprinting. Behav. Ecol. Sociobiol. **34**, 71–78. (10.1007/bf00175460)

[28] Emlen DJ. 1997 Alternative reproductive tactics and male-dimorphism in the horned beetle Onthophagus acuminatus (Coleoptera: Scarabaeidae). Behav. Ecol. Sociobiol. **41**, 335–341. (10.1007/s002650050393)

[29] Moczek AP, Emlen DJ. 2000 Male horn dimorphism in the scarab beetle, Onthophagus taurus: do alternative reproductive tactics favour alternative phenotypes? Anim. Behav. **59**, 459–466. (10.1006/anbe.1999.1342) 10675268

[30] Engqvist L, Taborsky M. 2016 The evolution of genetic and conditional alternative reproductive tactics. Proc. R. Soc. B **283**, 20152945. (10.1098/rspb.2015.2945) PMC4810835 26911960

[31] Fasel N, Saladin V, Richner H. 2016 Alternative reproductive tactics and reproductive success in male Carollia perspicillata (Seba’s short‐tailed bat). J. Evol. Biol. **29**, 2242–2255. (10.1111/jeb.12949) 27442591

[32] Kelly CD, L’Heureux V. 2021 Effect of diet and rearing density on contest outcome and settlement in a field cricket. Behav. Ecol. Sociobiol. **75**, 50. (10.1007/s00265-021-02990-w)

[33] Gross MR. 1991 Evolution of alternative reproductive strategies: frequency-dependent sexual selection in male bluegill sunfish. Phil. Trans. R. Soc. Lond. B **332**. (10.1098/rstb.1991.0033)

[34] MacColl ADC, Hatchwell BJ. 2002 Temporal variation in fitness payoffs promotes cooperative breeding in long‐tailed tits Aegithalos caudatus. Am. Nat. **160**, 186–194. (10.1086/341013) 18707485

[35] Riehl C, Strong MJ. 2019 Social parasitism as an alternative reproductive tactic in a cooperatively breeding cuckoo. Nature **567**, 96–99. (10.1038/s41586-019-0981-1) 30814729

[36] Knapp R, Hews DK, Thompson CW, Ray LE, Moore MC. 2003 Environmental and endocrine correlates of tactic switching by nonterritorial male tree lizards (Urosaurus ornatus). Horm. Behav. **43**, 83–92. (10.1016/s0018-506x(02)00018-1) 12614637

[37] Eberhard WG. 1982 Beetle horn dimorphism: making the best of a bad lot. Am. Nat. **119**, 420–426. (10.1086/283920)

[38] Partridge C. 2021 Making the best of a bad job. In Encyclopedia of evolutionary psychological science (eds TK Shackelford, VA Weekes-Shackelford), pp. 4660–4662. Cham, Switzerland: Springer International Publishing. (10.1007/978-3-319-19650-3_2696)

[39] Stearns SC. 1992 The evolution of life-histories. Oxford, UK: Oxford University Press. (10.2307/5403)

[40] Kirkwood TBL, Rose MR. 1991 Evolution of senescence: late survival sacrificed for reproduction. Phil. Trans. R. Soc. Lond. B **332**, 15–24. (10.1098/rstb.1991.0028) 1677205

[41] Rimbach R, Blanc S, Zahariev A, Pillay N, Schradin C. 2019 Daily energy expenditure of males following alternative reproductive tactics: Solitary roamers spend more energy than group-living males. Physiol. Behav. **199**, 359–365. (10.1016/j.physbeh.2018.12.003) 30521878

[42] Kanyile SN, Pillay N, Schradin C. 2021 Bachelor groups form due to individual choices or environmental disrupters in African striped mice. Anim. Behav. **182**, 135–143. (10.1016/j.anbehav.2021.10.005)

[43] Taborsky M. 1998 Sperm competition in fish: `bourgeois’ males and parasitic spawning. Trends Ecol. Evol. **13**, 222–227. (10.1016/s0169-5347(97)01318-9) 21238275

[44] Taborsky M. 2001 The evolution of bourgeois, parasitic, and cooperative reproductive behaviors in fishes. J. Hered **92**, 100–110. (10.1093/jhered/92.2.100) 11396567

[45] Sato T, Hirose M, Taborsky M, Kimura S. 2004 Size‐dependent male alternative reproductive tactics in the shell‐brooding cichlid fish Lamprologus callipterus in Lake Tanganyika. Ethology **110**, 49–62. (10.1046/j.1439-0310.2003.00944.x)

[46] Reznick D, Nunney L, Tessier A. 2000 Big houses, big cars, superfleas and the costs of reproduction. Trends Ecol. Evol. **15**, 421–425. (10.1016/s0169-5347(00)01941-8) 10998520

[47] Hurd PL. 2006 Resource holding potential, subjective resource value, and game theoretical models of aggressiveness signalling. J. Theor. Biol. **241**, 639–648. (10.1016/j.jtbi.2006.01.001) 16469335

[48] Sato Y, Rühr PT, Schmitz H, Egas M, Blanke A. 2016 Age‐dependent male mating tactics in a spider mite—a life‐history perspective. Ecol. Evol. **6**, 125–131. 7367–7374. (10.1002/ece3.2489) 28725404 PMC5513254

[49] Bel-Venner MC, Dray S, Allainé D, Menu F, Venner S. 2008 Unexpected male choosiness for mates in a spider. Proc. R. Soc. B **275**, 77–82. (10.1098/rspb.2007.1278) PMC2562406 17956845

[50] Cant MA, Johnstone RA. 1999 Costly young and reproductive skew in animal societies. Behav. Ecol. **10**, 178–184. (10.1093/beheco/10.2.178)

[51] Spong GF, Hodge SJ, Young AJ, Clutton‐Brock TH. 2008 Factors affecting the reproductive success of dominant male meerkats. Mol. Ecol. **17**, 2287–2299. (10.1111/j.1365-294x.2008.03734.x) 18410290

[52] Zhou S, Holmes MM, Forger NG, Goldman BD, Lovern MB, Caraty A, Kalló I, Faulkes CG, Coen CW. 2013 Socially regulated reproductive development: analysis of GnRH-1 and kisspeptin neuronal systems in cooperatively breeding naked mole-rats (Heterocephalus glaber). J. Comp. Neurol. **521**, 3003–3029. (10.1002/cne.23327) 23504961

[53] Swift-Gallant A, Mo K, Peragine DE, Ashley Monks D, Holmes MM. 2015 Removal of reproductive suppression reveals latent sex differences in brain steroid hormone receptors in naked mole-rats, Heterocephalus glaber. Biol. Sex Differ. **6**, 1–9. (10.1186/s13293-015-0050-x) 26693002 PMC4676092

[54] Montgomery TM, Pendleton EL, Smith JE. 2018 Physiological mechanisms mediating patterns of reproductive suppression and alloparental care in cooperatively breeding carnivores. Physiol. Behav. **193**, 167–178. (10.1016/j.physbeh.2017.11.006) 29730040

[55] Taborsky M, Cant MA, Komdeur J. 2021 Conflict. In The evolution of social behaviour, pp. 67–135. Cambridge, UK: Cambridge University Press. (10.1017/9780511894794.005)

[56] Schradin C, Lindholm AK, Johannesen J, Schoepf I, Yuen CH, König B, Pillay N. 2012 Social flexibility and social evolution in mammals: a case study of the African striped mouse (Rhabdomys pumilio). Mol. Ecol. **21**, 541–553. (10.1111/j.1365-294X.2011.05256.x) 21883591

[57] Cant MA, Vitikainen E, Nichols HJ. 2013 Demography and social evolution of banded mongooses. Adv. Stud. Behav. **45**, 407–445. (10.1016/b978-0-12-407186-5.00006-9)

[58] Cant MA, Nichols HJ, Thompson FJ, Vitikainen E. 2016 Banded mongooses: demography, life history, and social behavior. In Cooperative breeding in vertebrates: studies of ecology, evolution, and behavior (eds WD Koenig, JL Dickinson), pp. 318–337. Cambridge, UK: Cambridge University Press. (10.1017/CBO9781107338357.019)

[59] Cant MA. 2000 Social control of reproduction in banded mongooses. Anim. Behav. **59**, 147–158. (10.1006/anbe.1999.1279) 10640376

[60] Ancona S, Drummond H, Zaldívar-Rae J. 2010 Male whiptail lizards adjust energetically costly mate guarding to male–male competition and female reproductive value. Anim. Behav. **79**, 75–82. (10.1016/j.anbehav.2009.10.005)

[61] Ord TJ. 2021 Costs of territoriality: a review of hypotheses, meta-analysis, and field study. Oecologia **197**, 615–631. (10.1007/s00442-021-05068-6) 34716493

[62] Nichols HJ, Amos W, Cant MA, Bell MBV, Hodge SJ. 2010 Top males gain high reproductive success by guarding more successful females in a cooperatively breeding mongoose. Anim. Behav. **80**, 649–657. (10.1016/j.anbehav.2010.06.025)

[63] Sanderson JL, Wang J, Vitikainen EIK, Cant MA, Nichols HJ. 2015 Banded mongooses avoid inbreeding when mating with members of the same natal group. Mol. Ecol. **24**, 3738–3751. (10.1111/mec.13253) 26095171 PMC5008155

[64] Barry KL, Kokko H. 2010 Male mate choice: why sequential choice can make its evolution difficult. Anim. Behav. **80**, 323–333. 163–169. (10.1016/j.anbehav.2010.04.020)

[65] Edward DA, Chapman T. 2011 The evolution and significance of male mate choice. Trends Ecol. Evol. **26**, 647–654. (10.1016/j.tree.2011.07.012) 21890230

[66] Parker GA. 2006 Sexual conflict over mating and fertilization: an overview. Phil. Trans. R. Soc. B **361**, 235–259. (10.1098/rstb.2005.1785) 16612884 PMC1569603

[67] Johnstone RA, Cant MA, Cram D, Thompson FJ. 2020 Exploitative leaders incite intergroup warfare in a social mammal. Proc. Natl Acad. Sci. USA **117**, 29759–29766. (10.1073/pnas.2003745117) 33168743 PMC7703641

[68] Campos FA, Villavicencio F, Archie EA, Colchero F, Alberts SC. 2020 Social bonds, social status and survival in wild baboons: a tale of two sexes. Phil. Trans. R. Soc. B **375**, 20190621. (10.1098/rstb.2019.0621) 32951552 PMC7540948

[69] Nakagawa S. 2015 Missing data: mechanisms, methods, and messages. In Ecological statistics: contemporary theory and application, pp. 81–105. Oxford, UK: Oxford University Press. (10.1093/acprof:oso/9780199672547.003.0005)

[70] Green PA, Thompson FJ, Cant MA. 2022 Fighting force and experience combine to determine contest success in a warlike mammal. Proc. Natl Acad. Sci. USA **119**, e2119176119. (10.1073/pnas.2119176119) 35700363 PMC9231503

[71] Nichols HJ, Arbuckle K, Sanderson JL, Vitikainen EIK, Marshall HH, Thompson FJ, Cant MA, Wells DA. 2021 A double pedigree reveals genetic but not cultural inheritance of cooperative personalities in wild banded mongooses. Ecol. Lett. **24**, 1966–1975. (10.1111/ele.13833) 34176203

[72] Kéry M, Schaub M, Beissinger SR. 2012 Bayesian population analysis using winbugs a hierarchical perspective. Amsterdam, The Netherlands: Elsevier. See https://shop.elsevier.com/.

[73] Kellner K, Meredith M. 2024 jagsUI: a wrapper around ‘rjags’ to streamline ‘JAGS’ analyses. See https://cran.r-project.org/package=jagsUI.

[74] Marin X. 2021 ggmcmc: tools for analyzing MCMC simulations from Bayesian inference. See https://cran.r-project.org/package=ggmcmc.

[75] Freckleton RP. 2011 Dealing with collinearity in behavioural and ecological data: model averaging and the problems of measurement error. Behav. Ecol. Sociobiol. **65**, 91–101. (10.1007/s00265-010-1045-6)

[76] Mitchell J, Kyabulima S, Businge R, Cant MA, Nichols HJ. 2018 Kin discrimination via odour in the cooperatively breeding banded mongoose. R. Soc. Open Sci. **5**, 171798. (10.1098/rsos.171798) 29657784 PMC5882708

[77] Soulsbury CD, Halsey LG. 2018 Does physical activity age wild animals? Front. Ecol. Evol. **6**, 222. (10.3389/fevo.2018.00222)

[78] Taborsky M. 1994 Sneakers, satellites, and helpers: parasitic and cooperative behavior in fish reproduction. Adv. Study Behav. **13**, 222–227. (10.1016/S0065-3454(08)60351-4)

[79] Bourke AFG. 2014 Hamilton’s rule and the causes of social evolution. Phil. Trans. R. Soc. B **369**, 20130362. (10.1098/rstb.2013.0362) 24686934 PMC3982664

[80] Hamilton WD. 1964 The genetical evolution of social behaviour. I. J. Theor. Biol. **7**, 1–16. (10.1016/0022-5193(64)90038-4) 5875341

[81] Vitikainen EIK, Thompson FJ, Marshall HH, Cant MA. 2019 Live long and prosper: durable benefits of early-life care in banded mongooses. Phil. Trans. R. Soc. B **374**, 20180114. (10.1098/rstb.2018.0114) 30966878 PMC6460079

[82] Webster MS, Tarvin KA, Tuttle EM, Pruett-Jones S. 2004 Reproductive promiscuity in the splendid fairy-wren: effects of group size and auxiliary reproduction. Behav. Ecol. **15**, 907–915. (10.1093/beheco/arh093)

[83] Federico V, Allainé D, Gaillard JM, Cohas A. 2020 Evolutionary pathways to communal and cooperative breeding in carnivores. Am. Nat. **195**, 1037–1055. (10.1086/708639) 32469664

[84] Amundsen T, Forsgren E. 2003 Male preference for colourful females affected by male size in a marine fish. Behav. Ecol. Sociobiol. **54**, 89–96. 55–64. (10.1007/s00265-003-0593-4)

[85] Pizzari T, Cornwallis CK, Løvlie H, Jakobsson S, Birkhead TR. 2003 Sophisticated sperm allocation in male fowl. Nature **426**, 70–74. (10.1038/nature02004) 14603319

[86] Evans JP, Garcia‐Gonzalez F. 2016 The total opportunity for sexual selection and the integration of pre‐ and post‐mating episodes of sexual selection in a complex world. J. Evol. Biol. **29**, 2338–2361. (10.1111/jeb.12960) 27520979

[87] Holt WV, Fazeli A. 2010 The oviduct as a complex mediator of mammalian sperm function and selection. Mol. Reprod. Dev. **77**, 934–943. (10.1002/mrd.21234) 20886635

[88] Drewe JA. 2010 Who infects whom? Social networks and tuberculosis transmission in wild meerkats. Proc. R. Soc. B **277**, 633–642. (10.1098/rspb.2009.1775) PMC2842696 19889705

[89] Ophir AG. 2017 Navigating monogamy: nonapeptide sensitivity in a memory neural circuit may shape social behavior and mating decisions. Front. Neurosci. **11**. (10.3389/fnins.2017.00397) PMC5504236 28744194

[90] Cousseau L, van de Loock D, Githiru M, Vangestel C, Lens L. 2021 Female need for paternal care shapes variation in extra-pair paternity in a cooperative breeder. Behav. Ecol. **31**. (10.1093/beheco/arz215)

[91] Holmes MM, Goldman BD, Goldman SL, Seney ML, Forger NG. 2009 Neuroendocrinology and sexual differentiation in eusocial mammals. Front. Neuroendocrinol. **30**, 519–533. (10.1016/j.yfrne.2009.04.010) 19416733 PMC2748139

[92] Faulkes CG, Abbott DH, Jarvis JUM. 1991 Social suppression of reproduction in male naked mole-rats, Heterocephalus glaber. J. Reprod. Fertil. **91**, 593–604. (10.1530/jrf.0.0910593) 2013881

[93] O’Riain MJ, Bennett NC, Brotherton PNM, McIlrath G, Clutton-Brock TH. 2000 Reproductive suppression and inbreeding avoidance in wild populations of co-operatively breeding meerkats (Suricata suricatta). Behav. Ecol. Sociobiol. **48**, 471–477. (10.1007/s002650000249)

[94] Carlson AA, Young AJ, Russell AF, Bennett NC, McNeilly AS, Clutton-Brock T. 2004 Hormonal correlates of dominance in meerkats (Suricata suricatta). Horm. Behav. **46**, 141–150. (10.1016/j.yhbeh.2004.01.009) 15256303

[95] Woodroffe R, Rabaiotti D, Ngatia DK, Smallwood TRC, Strebel S, O’Neill HMK. 2020 Dispersal behaviour of African wild dogs in Kenya. Afr. J. Ecol. **58**, 46–57. (10.1111/aje.12689)

[96] Johnstone RA, Cant MA. 2010 The evolution of menopause in cetaceans and humans: the role of demography. Proc. R. Soc. B **277**, 3765–3771. (10.1098/rspb.2010.0988) PMC2992708 20591868

[97] Ellis S *et al*. 2022 Patterns and consequences of age-linked change in local relatedness in animal societies. Nat. Ecol. Evol. **6**, 1766–1776. (10.1038/s41559-022-01872-2) 36163259 PMC10423498

[98] Birch G. 2025 Alternative-tactics. GitHub. https://github.com/GrahamBirch/Alternative-tactics

[99] Cranmer K, Kreiss S. 2014 Decouple software associated to arXiv:1401.0080. Zenodo. (10.5281/zenodo.8475)

[100] Birch G, Nichols H, Mwanguhya F, Blount J, Cant M. 2025 Supplementary material from: Strategic use of male alternative reproductive tactics in cooperatively breeding banded mongoose groups. FigShare (10.6084/m9.figshare.c.7634292) PMC11858791 40012759

